# Towards a Scottish Safe Haven Federation.

**DOI:** 10.23889/ijpds.v7i3.1837

**Published:** 2022-08-25

**Authors:** Chuang Gao, Charlie Mayor, Sophie McCall, Katie Wilde, Christian Cole, Emily Jefferson

**Affiliations:** 1 Health Informatics Centre; 2 Glasgow Safe Haven; 3 Lothian/DataLoch Safe Haven; 4 DaSH, Aberdeen Centre for Health Data Science

## Objectives

Building upon the Scottish Safe Haven Network’s (SSHN) collaborative experience to develop an exemplar dataset on Scottish national level laboratory tests within the SSHN. To test the feasibility of a federated safe haven network in Scotland for clinical laboratory data using existing federation solutions.

## Approach

Considerations were given to the clinical tests that are being commonly used across Scotland. An investigation of laboratory data structure was conducted using SQL with the assistance of White Rabbit which captures the metadata information from each safe haven. Data heterogeneity or commonalities were investigated in detail in R. We examined the inter-relationships among clinical laboratories from different regions of NHS Scotland. A common data structure was proposed to facilitate the sharing of clinical test results. We investigated multiple existing federation solutions to streamline access to data across the SSHN.

## Results

A dataset of all laboratory tests for patients registered with the Scottish Health Research Register and Biobank (SHARE) from laboratory data sources within the network (Grampian Data Safe Haven, Health Informatics Center, Glasgow Safe Haven & Lothian/DataLoch Safe Haven) were developed. An open-source toolkit that includes SQL scripts to harmonise laboratory data from multiple safe haven and R scripts to conduct heterogeneity investigation to streamline the sharing and the analysis was created. We have shown a working model for federation within Scotland for the first time with the view to expand into more routine projects. The project has developed a systematic concept mapping of data available across the SSHN to aid cohort discovery.

## Conclusion

The exemplar national level laboratory data together with the toolkit will help provide researchers with information such as which data are available to access. It will also support and improve national-level research studies at a faster pace. The work has demonstrated the feasibility of a common process for federated data discovery and sharing across the SSHN.

